# Recent Trends in the Pre-Drying, Drying, and Post-Drying Processes for Cassava Tuber: A Review

**DOI:** 10.3390/foods13111778

**Published:** 2024-06-05

**Authors:** Ellyas Alga Nainggolan, Jan Banout, Klara Urbanova

**Affiliations:** 1 Department of Sustainable Technologies, Faculty of Tropical AgriSciences, Czech University of Life Sciences Prague, Kamýcká 129, 16500 Prague, Czech Republic; nainggolan@ftz.czu.cz (E.A.N.); urbanovak@ftz.czu.cz (K.U.); 2 Department of Bioprocess Engineering, Faculty of Biotechnology, Institut Teknologi Del, Jl. Sisingamangaraja, Sitoluama, Laguboti, Toba 22381, Indonesia

**Keywords:** cassava tuber, pre-drying, drying, post-drying, dried cassava products

## Abstract

Cassava tuber is an essential staple crop in tropical regions with versatile applications in the food, feed, and industrial sectors. However, its high moisture content and perishable nature necessitate efficient preservation methods to extend its shelf life and enhance its value. Pre-drying, drying, and post-drying processes play pivotal roles in maintaining the quality and usability of cassava products. This review comprehensively examines the current status and future directions in the pre-drying, drying, and post-drying processes of cassava tuber. Various pre-drying or pretreatment methods and drying techniques are evaluated for their impacts on drying kinetics and product quality. Additionally, challenges and limitations in achieving high-quality processing of cassava flour are identified. Future directions in cassava drying methods emphasize the integration of combined pre-drying and drying techniques to optimize resource utilization and processing efficiency. Furthermore, the adoption of advanced online measurement and control technologies in drying equipment is highlighted for real-time monitoring and optimization of drying parameters. The importance of optimizing existing processes to establish a comprehensive cassava industrial chain and foster the development of the cassava deep-processing industry is emphasized. This review provides valuable insights into the current trends and future prospects in cassava drying technologies, aiming to facilitate sustainable and efficient utilization of cassava resources for various applications.

## 1. Introduction

**Figure 1:**
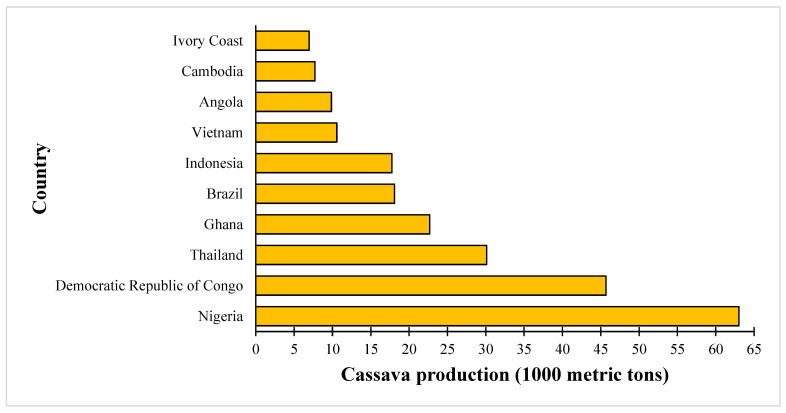
Leading cassava-producing nations across the globe in 2021.

Cassava, a staple tuber crop with a rich historical legacy of cultivation, thrives predominantly in tropical regions. In 2021, global cassava production reached an impressive 314 million metric tons [1], with various nations emerging as leaders in its cultivation, as illustrated in Figure 1. Currently, cassava plays a crucial role in the agricultural sector of tropical and underdeveloped nations. Parmar et al. [2] reported that cassava is a significant source of calories for over 800 million people in Africa, South America, and Southeast Asia. It plays a vital role in ensuring food security because it can produce approximately 10 tons per hectare, even in unfavorable soil conditions and with few inputs. Cassava tubers provide cost-effective pricing in comparison to other food commodities, and their economic worth can be augmented by means of processing and creating derivative products. Market Data Forecast [3] predicts that the global cassava flour market will reach USD 49.7 billion by 2028, with a compound annual growth rate (CAGR) of 6.4%.

Cassava serves not only as a source of carbohydrates but also harbors an array of nutrients such as ascorbic acid, carotenoids, calcium, potassium, iron, magnesium, copper, zinc, and manganese [4]. The presence of carbohydrates in cassava tubers enables their usage in diverse forms and applications [5]. Processing cassava can yield various food items that people can consume directly with simple processing or use as ingredients in food and beverages [6]. In addition, non-food industries make use of cassava, such as in alcohol manufacture, organic acid production, pharmaceuticals, paper manufacturing, and as additives. Traditionally, people often consume cassava tubers by boiling or cooking them, with variations in traditional processing methods observed across different areas and nations [7].

Cassava tubers may display indications of postharvest physiological deterioration (PPD) within 24–72 h of being harvested [8]. Cassava tubers typically have a moisture content of approximately 60–70%, carbohydrates ranging from 30% to 35%, and a protein content of 1% to 2% [9]. The elevated moisture content in cassava tubers impacts the advancement of cassava processing, necessitating prompt processing to generate intermediate products for the purpose of minimizing transportation expenses, prolonging shelf life, and enhancing storage capacity [10]. Drying is a preservation technique that aims to decrease the moisture content of food components, thereby prolonging their shelf life. Nevertheless, dried food components undergo certain modifications, including diminished nutritional value, changes in color, shrinkage, and differences in organoleptic quality, which directly affect consumer acceptability. Conventional drying methods for certain roots and tubers lead to a reduction in the number of bioactive substances present. This is caused by the loss of water, extended processing durations, exposure to oxygen and light, and processing temperatures surpassing 70 °C [11].

Prior to drying, implementing pre-drying process or pretreatment methods can improve permeability, expedite drying rates, stimulate enzymes, and inhibit oxidation in food materials [12]. Deactivating enzymes and reducing moisture using different pretreatment techniques, such as chemical, thermal, and physical approaches [13], are necessary to increase the amount of time tubers can be stored. Chemical precipitation methods can impede enzymatic browning reactions. Frequently employed chemicals for this purpose include sodium metabisulfite, sodium chloride, citric acid, and ascorbic acid [14]. Thermal treatments, such as blanching using hot water and steam, can deactivate polyphenol oxidase (PPO) enzymes. In addition to enzyme deactivation, this method significantly impacts texture, color, flavor, moisture content, and nutritional value [12].

Melese and Keyata [15] and Vera et al. [16] offer useful insights on pretreatment and drying procedures for different types of tubers. However, the cassava tuber drying sector still encounters numerous obstacles. Enduring challenges encompass the intricacy of drying systems, the expensive nature of equipment, and the technical complexities of operations. These obstacles have hindered the progress and acceptance of new drying and pretreatment methods. Therefore, it is crucial to carefully analyze both the economic feasibility and the quality of the output when drying cassava tubers. The objectives of this review were to (i) evaluate various pre-drying techniques and drying methods to understand their effects on drying kinetics and product quality; (ii) identify obstacles and limitations that prevent the production of high-quality cassava flour; and (iii) outline future directions for the pre-drying, drying, and post-drying processes of cassava.

## 2. Developments in Cassava Tuber Pre-Drying and Drying Research

Food drying, along with cooling and freezing, has been a longstanding preservation method. Its primary goal is to reduce moisture content in food products, thus enhancing their shelf life. Various factors influence the drying rate, including water transfer mechanisms, air properties, and material characteristics [17]. Pretreatment, commonly chemical or physical, significantly impacts both the drying process and material quality. One notable effect is the reduction in water activity (a_w_), which decreases microbial and enzymatic activities, enhancing preservation. Water activity is crucial for determining the availability of water for microbial and enzymatic reactions in food [18]. For staple foods like cassava, understanding the relationship between moisture content and water activity is essential for storage. Cassava sorption isotherms show how moisture content correlates with water activity, guiding optimal drying and storage conditions. Controlling water activity is vital in the drying process, as it affects the removal of water using various techniques. However, drying can impact the texture and quality of the original product, necessitating innovative pretreatment and drying technologies to preserve nutritional content, prevent chemical degradation, and maintain structure and texture [19,20]. By managing water activity during drying, it is possible to achieve preservation goals while preserving sensory and nutritional qualities. This knowledge underscores the importance of considering water activity in preservation strategies to ensure food quality and safety.

**Figure 2:**
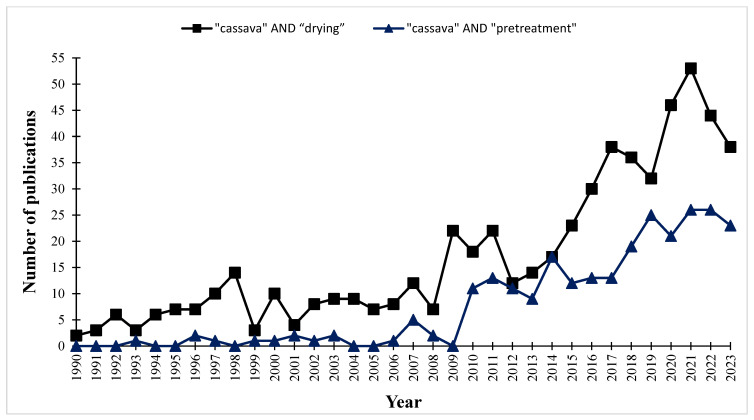
Pretreatment and drying technology trends for the period 1990–2023 as measured by the number of publications employing the following keywords: “cassava” AND “pretreatment”; “cassava” AND “drying”.

Conventional pretreatment and drying methods are being substituted by innovative techniques and hybrid processes, which involve the use of eco-friendly technology to promote sustainable food processing that must be implemented [21]. A survey of the Scopus database was undertaken to explore the progress in the adoption of current and improved procedures. The search was focused on the keywords “cassava + pretreatment” and “cassava + drying” within the time frame of 1990 to 2023. Figure 2 displays the quantity and pattern of publication data related to these keywords.

Additionally, safety risks associated with cassava consumption and the drying process are significant factors that warrant attention. Cassava contains cyanogenic glycosides, primarily linamarin, which can release cyanide upon hydrolysis [22]. Improper processing and inadequate drying techniques can lead to high cyanide levels in cassava products, posing health risks such as cyanide poisoning [23]. Moreover, microbial contamination during the drying process can result in the proliferation of harmful pathogens, leading to foodborne illnesses [24]. Therefore, comprehensive evaluations of pretreatment and drying methods for cassava must include assessments of cyanide levels, microbial safety, and overall food safety. Understanding the potential hazards and implementing appropriate control measures are crucial steps in ensuring the quality and safety of dried cassava slices or cassava flour throughout the value chain [25].

## 3. Advancements in the Research of Pre-Drying Techniques for Cassava Tubers

The following section presents a comprehensive overview of recent advancements in the study of pre-drying techniques and other potential techniques applicable to cassava tubers. Pre-drying, whether achieved through chemical or physical means, holds paramount importance in food processing, enhancing drying efficiency and elevating the overall quality of the final product. Physical pretreatment methods, including hot water blanching and steam blanching, play a pivotal role in preserving food quality by focusing on nutrient retention and preventing structural damage. On the other hand, chemical techniques such as acid treatment and sulfiting serve to deactivate enzymes, modify structure, and uphold flavor and nutritional content. Numerous pre-drying processes, particularly conventional pretreatment methods, are undergoing refinement to meet the demands of the lower-to-mid-tier food industry, which necessitates high-quality products with minimal nutrient loss, notwithstanding challenges such as operational inefficiencies and significant product alterations. Table 1 elucidates the impact of various pretreatment techniques on the drying characteristics and quality of dried cassava tubers.

### 3.1. Hot Water Blanching

A widely used pretreatment technique before drying is hot water blanching, which involves immersing fresh tubers in hot water at temperatures ranging from 70 to 98 °C for 5 to 15 min [31,32]. The main goal is to preserve the quality of the product by deactivating enzymes, eliminating bacteria, and extracting intercellular air from the tissues [33]. By changing the physical properties of the sample, such as making cell membranes more permeable and creating small cracks on the epidermis, hot water blanching speeds up the drying process [34,35]. However, conventional hot water blanching, despite its uncomplicated equipment and straightforward operation, has certain drawbacks. The pretreatment of cassava slices with hot water blanching at a temperature of 80 ± 2 °C for 5 min yields a lower whiteness index (ranging from 80.48 to 84.05) compared to pretreatment solely through soaking [36]. On the other hand, the degradation of food quality, namely the deactivation of oxidases, can result in alterations in flavor, appearance, and the depletion of heat-sensitive components. Furthermore, it is important to disregard the potential loss of soluble nutrients, such as carbohydrates, minerals, vitamins, sugars, and proteins, which may diffuse into the blanching water [37]. In addition, the process of hot water blanching has a negative impact on the texture and microstructure of the sample [29,38]. In the end, this approach produces a substantial amount of wastewater, which gives rise to environmental problems.

### 3.2. Steam Blanching

Pretreatment steam blanching is used to minimize the loss of nutrients, especially those that dissolve in water, and decrease the number of solid substances that dissolve in water, reducing waste. The selection of this technique is justified in comparison to hot water blanching, as it effectively preserves minerals and water-soluble constituents by minimizing the absorption effect [39]. Steam blanching combined with microwave vacuum drying significantly enhances drying efficiency and color retention of purple-fleshed sweet potato slices. The process reduced drying time to 7 min, achieving 7.5% moisture content. This method offers a rapid drying process with improved color preservation compared to hot water blanching [40]. Steam blanching significantly influences digestibility and β-carotene concentration in dried cassava, with drum-dried flours exhibiting higher digestibility, and air oven-dried samples showing elevated β-carotene levels [27]. However, there are obstacles related to the softening of tissues and changes in quality that occur due to extended heating duration. Additionally, uneven blanching effects occur due to steam condensation on the surface of the product during the initial stage of the process.

### 3.3. Sulfite Solution

The food industry has extensively utilized sulfidation, or the use of sulfur, to minimize browning during drying and to prevent degradation in quality during food processing and storage [41]. The standard method for conducting this procedure involves the utilization of sulfur dioxide gas or water-soluble sulfide salts, such as potassium metabisulfite (K_2_S_2_O), sodium metabisulfite (Na_2_S_2_O), and sodium hydrogen sulfide (NaHSO_3_). Sulfides, when used in low quantities, effectively inhibit enzymatic and non-enzymatic browning, as well as microbiological activity. Sulfite renders PPO inactive by engaging in a chemical interaction with quinones, hence impeding PPO’s functionality and diminishing the presence of oxygen [42]. The pretreatment of cassava slices with 4% Na_2_S_2_O resulted in a moisture content of 6.44% and a whiteness index of 93.87, with the determined activation energy for the process being 32.68 kJ/mol and the calculated effective moisture diffusivity as 6.23 × 10^−9^ m^2^/s [29]. In addition, sulfite functions as an antioxidant to inhibit the degradation of ascorbic acid and safeguard lipids, essential oils, and carotenoids against oxidative harm during processing. This offers the benefits of keeping color, reducing damage, and maintaining some nutritional properties.

Sulfidation can result in the depletion of water-soluble nutritional components, the development of undesirable odors, the alteration of textures to a softer state, and the existence of chemical residues on treated food items [43,44]. While sulfidation is still successful in maintaining the color of products, its drawbacks make it less preferable, particularly in the growing trend of organic food production.

### 3.4. Acid Solution

Acid pretreatment is a prevalent technique that improves the caliber of agricultural commodities by inactivating enzymes, augmenting pigment durability, and altering texture. The basis of this process involves adjusting the pH, inhibiting the activity of polyphenol oxidase, and decreasing color changes caused by enzymatic processes. Acidic substances with a pH between 3.0 and 7.0 are advantageous for retaining the stability of pigments, and also help maintain the texture of the product due to their chelating capabilities. Citric acid, a prevalent organic acid, functions as both an anti-browning agent and an efficient texture modifier for fruits and vegetables [45]. The pretreatment of cassava slices with 4% citric acid resulted in a moisture content of 6.53% and a whiteness index of 93.49, with determined activation energy for the process at 32.83 kJ/mol, and an effective moisture diffusivity of 6.32 × 10^−9^ m^2^/s [29]. Although acid pretreatment offers advantages, it also has disadvantages, including the depletion of water-soluble nutrients and the deterioration of acid-sensitive pigments. Nevertheless, this method continues to be valuable for improving the quality and longevity of agricultural goods.

### 3.5. Ultrasonic Field

Ultrasonication is a pretreatment technology that uses high-frequency ultrasonic waves to create, enlarge, and burst bubbles in a liquid medium, a process called cavitation [46]. The pretreatment method, which produces a large number of bubbles, enhances the transfer of mass in food items by utilizing both direct mechanisms (inertial flow and the “sponge effect”) and indirect mechanisms (creation of microchannels) [47,48]. The application of ultrasonic treatment at ambient temperature allows for the preservation of thermally delicate components in food [49]. Ultrasonic-assisted techniques, as an emerging treatment, have garnered significant interest in agricultural product research due to their capability to facilitate the drying process, increase drying rates, and enhance product quality by modifying the microstructure of plant tissue [30,31,47,50].

Ultrasound pretreatment of potatoes results in improved color and texture attributes, while it also causes more damage to the cell structure compared to the control group [51]. While it does minimize processing time and preserve product quality, its downside lies in causing structural harm to plant tissues, leading to a reduction in phytochemical content. Technological limitations encompass factors such as restricted capacity to scale up, the intensity of cavitation, and the requirement for coupling media (such as gel, water, or oil), which present difficulties for implementing large-scale applications in the food industry [52]. Possible solutions entail the advancement of non-contact ultrasonic methods that utilize air as a coupling medium to tackle practical challenges and fulfill the requirements of large-scale companies [53].

### 3.6. Alternative Prospective Pre-Drying Techniques for Cassava Tubers

**Figure 3:**
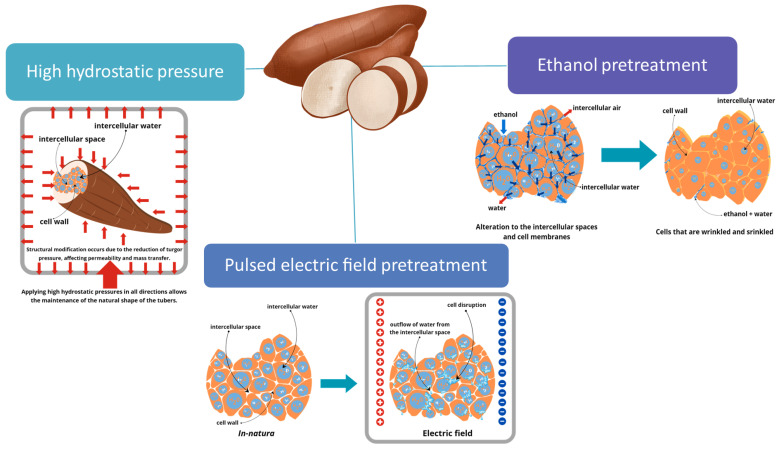
The effect of ethanol solution, pulsed electric field and high hydrostatic pressure pretreatment techniques on tubers.

The following section introduces alternative methods for preparing cassava tubers prior to drying, highlighting advancements in their processing techniques. While some approaches show promise in enhancing the efficiency of cassava tuber pretreatment, others may pose challenges such as complexity, high costs, and the need for specialized equipment and expertise. In pursuit of optimizing treatment duration, product quality, and energy consumption, emerging pretreatment systems like pulsed electric field have been explored as potential alternatives to conventional methods such as ethanol pretreatment. These systems often combine multiple pretreatment techniques, leveraging the benefits of each approach. However, selecting the most suitable methods and components requires a comprehensive understanding of the specific requirements and challenges associated with cassava tuber processing. Carvalho et al. [17] have documented various pretreatment technologies for drying roots and tubers, including the effects of ultrasound, pulsed electric field, high hydrostatic pressure and ethanol pretreatment on tubers. Figure 3 illustrates the mechanisms of ethanol solution, pulsed electric field, and high hydrostatic pressure pretreatment techniques applied to tubers.

#### 3.6.1. Ethanol Solution

The use of ethanol solution as a pretreatment in drying research is becoming more common. It has been found to be effective in different applications, including immersion, spraying, and atmospheric modification of agricultural products [54,55]. The process entails alterations in the structure of plant tissues when exposed to ethanol, such as wilting, thinning of cell walls, and disruption of cellular organization. These changes ultimately facilitate the transfer of mass [56]. Figure 3 displays the effects of ethanol pretreatment on tubers. Applying ethanol pretreatment to carrots and potatoes can effectively decrease drying time by up to 50% and significantly reduce energy usage in carrot drying by 56% [57,58].

While the use of this method is successful in decreasing the time it takes for carrots and potatoes to dry, it does not consistently lead to improvements in the overall process. Rojas and Augusto [59] observed that infrared drying of potatoes treated with ethanol negatively impacted their ability to rehydrate due to significant structural alterations. Variables such as the duration of pretreatment and the thickness of the sample have an impact on the extent to which ethanol may penetrate, which is essential for its efficacy, particularly in solid items such as roots and tubers. Although ethanol shows promise, additional research is necessary to maximize its use in improving the drying quality of different types of vegetables, especially roots and tubers.

#### 3.6.2. Pulsed Electric Field

Pulsed electric field (PEF) technology is a method that uses short, intense electric pulses to treat food. This treatment can be applied to liquid, semi-solid, or solid food by immersing the food in a liquid before applying the pulses. The treatment is applied between two electrodes [60]. The utilization of PEF involves the inactivation of microbes and enzymes at room temperature while simultaneously maintaining the quality characteristics of food such as color, flavor, texture, and nutritional content [61]. This process improves the ability of substances to pass across the cell membrane and breaks down tissues, hence increasing the movement of substances in plant materials [62]. The impacts of pulsed electric field pretreatment on cassava tubers are illustrated in Figure 3. PEF has been successfully used in agricultural products to enhance the drying process by increasing the permeability of cell membranes and protecting food quality by deactivating enzymes [63]. Liu et al. [64] demonstrated that PEF pretreatment in conjunction with vacuum drying effectively reduces the drying process duration, mitigates color alterations, and improves carrot rehydration ability.

Nevertheless, PEF has significant limitations, such as its incapacity to inactivate enzymes in specific circumstances, resulting in cell harm and tissue softening, as well as the considerable expenses associated with the required equipment [65]. Furthermore, the presence of food ingredients that have large particles or exhibit strong electrical conductivity can present difficulties [66].

#### 3.6.3. High Hydrostatic Pressure

High hydrostatic pressure (HHP) technology is a type of pretreatment that uses high-pressure shock waves (100–800 MPa) conveyed by water to treat materials at precise durations and temperatures [67,68]. The application of pretreatment in food products leads to cell permeabilization, promotes diffusion, and increases drying rates. The utilization of HHP on cocoyam, Peruvian carrot, and sweet potato has shown decreased drying durations and limited degradation of quality, as indicated by changes in texture, higher rates of moisture transfer, and improved rehydration abilities [69]. Nevertheless, the main obstacles to implementing HHP are the exorbitant expenses associated with the equipment, the limited processing capacity, and the unpredictability of enzyme deactivation [65]. According to Jermann et al. [70], the commercial adoption of HHP in the food industry necessitates further research and development to address structural issues and the permeation of pressure mediums into food products.

## 4. Advancements in the Research of Drying Methods for Cassava Tubers

The following section offers a comprehensive overview of the latest advancements in research pertaining to the drying of cassava tubers, as well as other potential techniques. Drying, a method employed for food preservation since ancient times, is explored in detail here, with a focus on various conventional and emerging methods used for drying cassava tubers. These methods encompass sun drying, solar drying, hot air oven drying, vacuum drying, freeze drying, fluidized bed drying, solar-assisted drying, and microwave-assisted drying. Understanding the strengths and limitations of each technique is crucial for selecting the most suitable approach for specific application scenarios. Table 2 outlines the impact of different drying methods on the drying characteristics and quality of cassava tubers.

Drying significantly reduces anti-nutritional factors in cassava. Dahal and Tamang [78] reported that both sun-drying and cabinet drying resulted in substantial reductions in cyanide, tannin, oxalate, and phytate levels compared to raw cassava. Sun-drying, particularly, achieved greater reductions, with cyanide levels decreasing by 56.79% (to 37.29 mg/kg) and tannin, oxalate, and phytate levels decreasing by 94.2%, 79%, and 81.73%, respectively. Cabinet drying also showed reductions, though to a lesser extent, with cyanide decreasing by 23.69% (to 65.84 mg/kg) and tannin, oxalate, and phytate levels decreasing by 12.31%, 37.2%, and 41.34%, respectively. Nebiyu and Getachew [79] similarly reported that sun-drying cassava chips treated with a 24-h water soaking significantly reduced the total hydrogen cyanide content in cassava flour by over 80%.

Mechanical and natural drying of cassava influence anti-nutritional cyanogenic glucoside levels, with sun drying generally leading to better degradation due to optimal enzyme activity and lower temperatures, while oven drying retains more cyanide, especially at higher temperatures and with thinner chips, making drying methods less effective for detoxification of cassava high in initial cyanogen [80]. Drying fresh, peeled cassava chips significantly reduced total cyanogen content, with the extent of reduction influenced by chip size, temperature, and drying time; notably, thicker chips exhibited a higher reduction due to slower drying rates allowing more linamarase–glucoside interaction [81].

Pre-drying fermentation plays a significant role in diminishing cyanide levels in cassava, thereby enhancing linamarase activity [78,82]. This methodology proves highly effective in reducing cyanide content to levels below the recommended threshold prior to the drying process, a departure from conventional techniques. Moreover, the conditions during solar drying exert a notable influence on cyanide retention, implying a versatile strategy to alleviate the presence of anti-nutritional factors in dried cassava [82].

### 4.1. Sun Drying

Cassava tubers can be dried either by exposing them directly to sunlight or by placing them on bamboo racks with a plastic sheet underneath. Smallholder farmers widely adopt sun drying due to its straightforwardness and cost efficiency. To expedite the drying process of cassava tubers, it is common practice to decrease their size, hence increasing the exposed surface area [83]. According to Alamu et al. [71], the moisture content of dried cassava tubers can reach a level of 6.55% ± 0.07 by using sun drying. This can be done by maintaining drying settings of 18 to 25 °C and humidity levels between 60 and 75%. Nevertheless, the utilization of open-air drying methods might result in the introduction of impurities and a rise in moisture levels, even after achieving a state of equilibrium moisture content [84]. Alamu et al. [71] also observed that sun drying cassava has the disadvantage of causing the tubers to change color, with the largest ΔE values (16.37–24.78) compared to oven drying. Managing drying parameters in sun drying is a difficult task. Although this process is inexpensive, it lacks reliability in preserving the quality of dehydrated tubers.

### 4.2. Solar Drying

Solar drying is a more advanced drying technique than sun drying, where the drying process takes place inside a mechanical structure that harnesses solar energy [85]. Its operational simplicity and economic affordability have led to its increased appeal, especially among small-scale farmers. Although open-air drying under sunlight is cost-effective and requires minimal work, it has several obstacles. These include extended drying durations, the risk of contamination from stones and debris, and exposure to exhaust emissions on the roadside, which can compromise the nutritional value of the product. According to Suherman et al. [86], solar drying, examined on seaweed and cassava starch, proves effective in reducing moisture content, influenced by drying time and temperature, indicating its viability as a feasible method for efficient drying and maintaining product quality. The yams, which were subjected to solar drying, achieved an equilibrium weight between 390 and 480 min, with drying rates ranging from 10% to 20% per hour [87]. Despite the impact of environmental elements such as temperature, sun radiation, and rainfall on solar drying, several system advancements have been implemented to tackle these challenges.

### 4.3. Hot Air Oven Drying

The process of hot air oven drying is based on the idea of mechanical air convection, where an electrically powered exhaust fan is used to regulate the internal temperature of the drying chamber. The use of hot air ovens for drying yellow cassava yields moderate digestibility levels, ranging from 60.3% to 70.4%, whereas drum drying shows higher digestion rates, ranging from 69.4% to 79.7%, and solar drying yields comparable results, ranging from 60.4% to 70.7%, with hot air oven drying also demonstrating higher concentrations of β-carotene compared to alternative drying techniques [88]. Famurewa et al. [89] found that using a hot air oven at a temperature of 60 °C for a duration of 14 h resulted in cassava tubers being dried more quickly and with a lower moisture content compared to traditional methods like sun drying. The process of drying yams using a hot air oven results in the highest levels of protein, ash, fiber, and carbohydrates, as well as minerals like calcium (Ca), magnesium (Mg), and phosphorus (P). However, the moisture content is generally lower compared to that obtained with sun drying methods [90]. The use of hot air oven drying is favorable due to its cost-effectiveness in comparison to vacuum and freeze-drying methods while still producing higher-quality products than sun drying [71,91].

### 4.4. Vacuum Drying

Vacuum drying is widely used in the food, agricultural, and pharmaceutical industries. It involves drying at low temperatures using steam, hot air, or inert gas. This is done by passing the gas through empty shelves or over hot plates in a high-pressure vacuum environment [92]. Rashid et al. [93] assert that vacuum drying of potatoes is a superior and efficient drying approach when compared to conventional methods like sun drying or open-air drying. This technique expedites the dehydration process, resulting in superior-quality goods and maintaining optimal nutritional value in the dehydrated potatoes. Yang et al. [94] found that vacuum drying of potatoes decreases moisture content, alters starch properties favorably, and reduces oil absorption by forming protective layers, although microstructural changes observed via SEM negatively impact oil absorption, providing insights for improving fried potato quality and controlling oil content. Although vacuum drying has some advantages, it is still very expensive and time-consuming compared to other drying methods mentioned in prior studies. Another obstacle is the ability to scale up this drying process to meet commercial demands.

### 4.5. Freeze Drying

Freeze drying, a technique used to preserve heat-sensitive and biologically based food products for extended periods of time, relies on sublimation as an essential step. Alamu et al. [71] found that freeze drying with a run collector working condition at a temperature of −41 °C for 48 h resulted in sliced cassava tubers with a higher moisture content (9.77 ± 0.01) compared to oven drying (6.22 ± 0.02). Nevertheless, the color alterations observed in the dehydrated cassava tubers using freeze drying exhibited the least magnitude when compared to sun drying and oven drying methods [71]. Various elements, such as the thermal conductivity of the heating plate and the temperature difference between the sample and the air, influence the heat transmission mechanism in freeze drying. Li et al. [95] found that freeze drying preserves appearance, color, microstructure, and active components of *Bletilla striata* tubers effectively compared to other drying methods, making it the most suitable option for preserving quality and active components, beneficial for medicinal and tonic functions. Freeze drying after high shear mixing (HSM) at varying speeds enhances total dietary fiber content in cassava starch, reaching up to 14.66% at 12,000 rpm, thus improving its potential as a source of resistant starch [96]. In general, the freeze-drying technique for cutting tubers has been thoroughly investigated and proven to be highly effective in creating superior dried products. Nevertheless, this technology has certain disadvantages, such as substantial investment and operational expenses, extended drying durations, and the difficulty of expanding operations.

### 4.6. Fluidized Bed Drying

Fluidized bed drying is based on the principle of sample fluidization, in which moist particles maintain a fluidized state through continuous interaction with a heated surface or hot air blown over them. It is frequently used to remove moisture from wet powders and solid capsules or particles. This technique enables effective heat and mass transmission, leading to quick drying durations, rapid drying rates, effectiveness, and consistent condensation [97]. Furthermore, Famurewa and Emuekele [98] reported that fluidized bed drying at 70 °C and an air velocity of 2.75 m/s significantly reduces the cyanide content in cassava chips. The moisture content of potatoes decreases as the drying air temperature increases while utilizing fluidized bed drying, regardless of whether the potatoes are cuboid or cylindrical in shape [99]. According to Lozano-Acevedo et al. [100], higher temperatures and higher concentrations of citric acid result in more significant alterations in color. Additionally, decreasing the amount of water and water activity in potato samples dried using fluidized bed drying also contributes to these color changes. Fluidization drying systems have wide acknowledgement and effective implementation on an industrial level. Although the distributor placement has certain benefits, such as its static nature and low particle velocity, it can also lead to issues with energy consumption and air circulation. These problems can have a negative impact on drying rates, causing cracking and damage to occur [101].

### 4.7. Solar-Assisted Drying

The solar-assisted heat pump dryer (SAHPD) emerges as a promising technology offering a sustainable solution for drying biomaterials like cassava, thereby mitigating postharvest losses. SAHPDs are favored over other drying methods due to their economic efficiency, reliability, and ability to produce high-quality dried products [102]. Unlike passive solar dryers reliant solely on sunlight or other energy-intensive drying methods, SAHPDs can operate effectively even in the absence of direct sunlight, ensuring consistent product quality throughout the day [103,104]. Loemba et al. [105] reported the significant benefits of utilizing heat pumps in agricultural product drying. Employing heat pumps notably reduced drying time, enhanced specific moisture extraction, and lowered energy consumption. In the case of cassava, the combined use of solar drying (SD) and SAHPD decreased the mass from 30.8 kg to 17.4 kg within 13 and 9 h, respectively, at average temperatures of 40 °C and 45 °C. The moisture content of cassava decreased from 61% to 10.5%, with corresponding mass flow rates [84]. Furthermore, the study reported average thermal efficiencies of 25.6% for SD and 30.9% for SAHPD, along with respective average drying rates and specific moisture extraction rates. The integration of solar-assisted heat pump drying technology exhibits notable improvements in drying characteristics, offering reduced drying times, enhanced energy efficiency, and improved product quality, particularly exemplified in the drying of cassava.

### 4.8. Microwave-Assisted Drying

Conventional microwave drying has a faster drying rate and low energy consumption compared to most other conventional drying methods for drying roots and tubers [17]. Nevertheless, this drying method experiences product non-uniformity as a result of an unequal distribution of the electromagnetic field within the drying chamber. The non-uniform intensity of the electric field, along with insufficient methods to dissipate excessive thermal energy from the product, can cause localized overheating, leading to subpar product quality and tissue harm. In order to overcome these restrictions, researchers have investigated different hybrid microwave drying approaches, including the combination of microwave drying with other methods, such as microwave-vacuum drying (MWVD) [106], microwave-assisted freeze drying (MWFD), and microwave-enhanced spouted bed drying (MWSD), for a range of industrial purposes. According to Regier et al. [107], carrot drying using MWVD required less than 2 h, while conventional drying took 4.5–8.5 h. MWVD has also been observed in the processing of yams [108], potatoes [109], and beets [110]. In a comparison of MWVD, MWFD, and MWSD on carrots, Yan et al. [111] discovered that MWSD produced the most uniform color and the maximum drying rate at 3.5 W/g. Additional research conducted on the drying of potatoes and yams utilizing MWFD also demonstrated enhancements in the drying process, resulting in reduced drying time [108].

### 4.9. Alternative Prospective Drying Methods for Cassava Tubers

This section provides a thorough examination of various research efforts in the field of food drying, with a specific emphasis on novel techniques that can be utilized for drying cassava tubers. Although several drying procedures have proven to be effective in creating dry materials, many of them are complex, expensive, and require specialized equipment and knowledge. In addition, the conversation explores hybrid drying techniques, which researchers have investigated as possible options that provide benefits in terms of drying time, product quality, and energy efficiency compared to traditional methods. Hybrid drying systems usually combine different drying methods to make use of the benefits of each approach. This requires a thorough assessment of criteria and difficulties in order to choose the most suitable techniques and components. In addition to these approaches, various other procedures have been examined for the purpose of drying cassava slices. However, it is crucial to conduct further study and make necessary improvements in order to enhance these techniques. These diverse methods contribute to the ongoing examination and improvement of cassava drying processes, highlighting the significance of continued study and improvement efforts.

#### 4.9.1. Infrared-Assisted Drying

Infrared radiation (IR) drying and IR-assisted drying utilize electromagnetic radiation with wavelengths that are longer than those of visible light [21,112]. During the drying process, the material being dried absorbs infrared (IR) energy, resulting in the elimination of moisture and the heating of the substance. This causes the material to dry from the outer surface to the inner part [112]. The heat source can be powered by either electricity or gas, and the radiation strength can be modified to attain the desired degree of drying. Research suggests that infrared (IR) drying improves the quality of dried items by reducing drying time and enhancing rehydration behavior [113]. Nevertheless, the excessive use of electricity can lead to undesirable outcomes in the quality of the end product. This includes reduced ability to rehydrate and noticeable alterations in color for sweet potatoes [114,115], as well as diminished retention of ascorbic acid in carrots [116].

The integration of convective drying and infrared drying techniques yields accelerated drying durations, decreased energy usage, and enhanced mass transfer rates while drying sweet potatoes [115]. However, it is crucial to apply hybrid drying methods correctly in order to prevent oxidative enzymatic activity and the loss of important components in sweet potatoes [117]. Far infrared radiation (FIR) drying has a greater ability to penetrate materials compared to near infrared radiation (NIR). It also has a stronger effect on materials with high moisture content, leading to a higher preservation of antioxidant properties in seaweed compared to freeze drying [118]. In addition, infrared (IR) can also assist in freeze drying processes (IRFD), resulting in reduced drying time and improved quality, as demonstrated in research conducted on sweet potato slices [119]. However, the untapped potential of using IR drying for sliced cassava tubers has not been thoroughly investigated.

#### 4.9.2. Ultrasound-Assisted Drying

The incorporation of ultrasonic technology into the HAD process is considered essential to overcome the constraints. Ultrasound technology has potential for improving the HAD process by decreasing the resistance to moisture diffusion within and increasing the transfer of moisture internally [120]. Multiple forms of ultrasound technology, such as air ultrasound and contact ultrasound [36], have been proven to accelerate drying periods and reduce the resistance of moisture transfer throughout the drying process. Mulet et al. [121] state that during the process of drying, mechanical waves travel through the air and food products, causing small waves to form at the surface of the solid. This results in a decrease in external resistance, an increase in mass transfer, and a faster diffusion process. The utilization of ultrasound during the drying process for carrots led to a notable decrease in drying time by 22% when using a power of 200 W [122]. Carcel et al. [123] found similar results, showing that carrots dried 30% faster when subjected to processing conditions of 75 W power and 21.7 kHz frequency. In contrast, the utilization of ultrasound-assisted drying on potatoes resulted in a reduction of drying time by as much as 47.7% when using 60 W power. However, this process did modify the structure of the dried potatoes [124]. The impact of ultrasound differs among various matrices, such as roots and tubers, due to inherent variables including tissue porosity and cell structure compactness. Hence, it is crucial to understand the essential attributes of the food prior to utilizing ultrasound techniques in order to achieve dried food with enhanced quality and reduced drying time.

#### 4.9.3. Refractance Window Drying

Refractance window drying (RWD) is a novel drying method that uses thin transparent plastic sheets as a “window” to generate heat through infrared radiation, drying substances as flakes or powder [125]. Subsequently, circulated hot water is used to heat the materials from below. This drying method provides clear benefits, particularly in generating high-quality dried products in terms of color, bioactive components, and overall attractiveness. The drying process for this product is characterized by short duration and low temperatures (30–70 °C), which effectively preserve its nutritional content, including vitamins and antioxidants, as well as its color [126]. Heat transfer takes place through three modes, enabling fast processing, and drying stops once it reaches the point of dry equilibrium [127]. More precisely, RW technology exhibits superior thermal efficiency and cost-effectiveness, as stated by Bernaert et al. [126]. The utilization of RWD has been expanded to encompass a wide range of root crop goods, including potatoes [128]. Duarte-Correa et al. [129] found that potato flour has water-holding capacities ranging from 0.35 to 4.68 g/g and oil-holding capacities ranging from 0.80 to 1.73 g/g. This demonstrates that RW drying is capable of producing powders with reliable and uniform technological properties. As a result, RWD shows great potential for use in various applications within the food industry. Ueda et al. [130] found that the functional, nutritional, and sensory characteristics of the powder produced are impacted by various processing variables, such as water temperature, drying duration, and sample thickness. While RW drying has several benefits, it is important to note that there are potential disadvantages, such as the requirement for meticulous regulation of processing settings and the possibility of impacting the characteristics of the dried product.

## 5. Post-Drying Operations for Dried Cassava Tubers

Cassava, a staple crop in numerous tropical and subtropical regions, holds significant economic importance by serving both as a fundamental food source and as a primary income generator for farmers [131]. Enhancing cassava processing techniques, particularly drying methods, can improve food security, promote sustainable livelihoods, and strengthen social bonds within communities [132]. Notably, advancements in cassava processing, particularly in the realm of drying techniques, have markedly bolstered its market viability and economic worth. This improvement stems from the successful reduction of moisture content to levels compatible with most market requirements [133,134], rendering cassava products stable for extended periods of storage [135].

Cassava chips and cassava flour are the predominant products used in cassava tuber drying applications. The study conducted by Oladejo et al. [30] demonstrated that employing ultrasound in distilled water and an osmotic solution as pretreatment for convective drying of cassava tuber slices might result in significant reductions in energy consumption, time, and overall costs associated with the drying process. Pornpraipech et al. [136] conducted drying research that demonstrated how the shape and weather circumstances affect the drying process of cassava tuber slices. Veeramanipriya and Sundari [137] conducted drying research on cassava tuber slices using a hybrid photovoltaic thermal solar dryer equipped with an evacuated tube collector as the drying processing instrument. The study findings indicated that hybrid drying outperformed sun drying in terms of the dimensions, form, hue, visual aspect, texture, and overall excellence of the dehydrated cassava tuber slices. Assessing modified starch, specifically in flour materials, is essential, particularly due to its involvement in the preservation process, which includes drying [57]. Table 3 presents various examples of diverse food products primarily composed of flour that utilize cassava flour.

Effective packaging and storage are essential actions after the drying process to preserve the quality of food products. The presence of carbohydrates, proteins, and lipids in cassava tubers contributes to both enzymatic and non-enzymatic reactions that occur during the storage of dried cassava tubers. Endogenous processes and microbial activity generate a range of aromatic scents and soft textures through biochemical interactions. Cassava tuber slices may harbor harmful microorganisms, such as bacteria, viruses, yeast, and molds. Alamu et al. [71] reported that cassava flour of superior quality is characterized by low ash content and low moisture content.

The storage conditions for cassava flour are a crucial component that directly impacts the quality of the flour for its eventual utilization. Utilizing acceptable packaging materials, such as plastic buckets, jute, low-density polyethylene bags, sacks, and paper bags, is recommended for flour goods [145]. Ogiehor and Ikenobomeh [146] conducted a study where they used different materials, including low-density polyethylene bags, Hessian bags, high-density polyethylene bags, and plastic buckets, to package stored garri at a temperature of 30 °C for 24 weeks. The study found that all packaging types resulted in an increase in moisture content in the samples. Ensuring the ideal temperature and relative humidity is essential for maintaining the quality of products. Inadequate packaging materials can lead to a decline in the quality and shelf life of flour [147]. The choice of packaging materials has an impact on the stability of physicochemical and microbiological properties during storage. According to Opara et al. [147], plastic buckets resulted in the least color change in cassava flour, plastic packaging resulted in the largest amount of carotenoid content, and paper bags resulted in the lowest counts of aerobic mesophilic bacteria and molds in cassava flour.

Efficiently optimizing the drying process is crucial for guaranteeing the exceptional quality of cassava flour. Thus, it is crucial to exert meticulous control over temperature, accurately determine when drying is complete, and maximize drying efficiency. This methodical approach ensures that the used process optimizes the quality of cassava flour. The implementation of cutting-edge measurement and control approaches [148,149], such as low-field nuclear magnetic resonance (LF-NMR) technology, is crucial in achieving this goal. LF-NMR allows for the measurement and tracking of moisture levels during the drying process for cassava tubers. This enables the accurate prediction of when the drying is complete and ensures the production of high-quality cassava flour. These measures not only improve the quality of processing but also reduce costs. To achieve high-quality cassava flour manufacturing, it is essential to optimize the drying process and incorporate LF-NMR technology. Afterwards, the resultant superior cassava tubers can be used together with other items to improve their nutritional content, promoting high-quality growth.

## 6. Conclusions

The increasing demand for cassava and its derivatives requires the implementation of effective pretreatment and drying methods to guarantee high quality and meet the demands of the market. Although these processes are important, there is minimal study on innovative methods compared to those for other dietary components. Future research should prioritize the development of more-efficient arrangements to maintain the nutritional and physicochemical properties, with a specific emphasis on non-thermal pretreatment and low-temperature drying techniques. Furthermore, it is imperative to evaluate the influence of different factors on the quality of cassava flour and develop methods for ensuring quality control and monitoring throughout the pre-drying, drying, and post-drying stages.

The focus should be on implementing effective pretreatment, drying techniques and post-drying operations to decrease operational costs, eliminate waste, and improve profitability for cassava tuber drying facilities. It is important to prioritize the incorporation of pretreatment and drying technologies into small-scale businesses. This can be achieved by focusing on practical challenges through education and awareness initiatives. Furthermore, it is important to create automated technologies for the purpose of process control and quality assessment, while also ensuring a harmonious equilibrium between capital investment and technical advancement. Future study should focus on prioritizing sustainable drying processes for cassava products, taking into account the environmental consequences and societal advantages. Integrating computer-based techniques and artificial intelligence into process control and quality assessment systems is crucial for further enhancing cassava pre-drying, drying, and post-drying technologies. Promising research fields are the enhancement of non-invasive methodologies and the incorporation of artificial intelligence into process management and product evaluation.

## Figures and Tables

**Table 1:** Pre-drying techniques adopted for the drying of cassava tuber.

Pre-Drying Technique	Pre-Drying Condition	Drying Method	Major Observations	References
Hot Water Blanching	70 °C for 6 min Sample-to-water ratio 1:5 (*w*:*w*)	Microwave–hot air drying	Pre-drying and drying process had uniform effects on cassava regardless of cultivar variation. Starch changes during cooking did not affect drying kinetics, resulting in consistent texture and sensory scores.	[26]
Steam Blanching	110 °C for 5 min	Solar drying Hot air oven drying Drum drying	Pre-drying with steam blanching followed by drum drying yields higher digestibility and preferable sensory attributes, while hot-air oven drying enhances β-carotene concentration. Cultivar and drying method influence flour characteristics, highlighting the potential of drum drying for optimal product quality.	[27]
Sulfite Solution	0.3% sodium metabisulfite solution for 7 min	Flash drying	Pre-drying with sodium metabisulfite followed by flash-drying enhances rheological properties and water absorption capacity of cassava flour, making it suitable for formulations requiring good pasting quality and moderately high gel strength.	[28]
Acid Solution	0.3% citric acid solution for 7 min	Cabinet drying	Citric acid-treated flour exhibits inferior rheological traits and water absorption capacity compared to sodium metabisulfite-treated counterparts.	[28]
Combined	1.31% citric acid and 1.03% sodium metabisulfite for 20 min Steam blanching at 80 °C for 1.01 min.	Hot-air oven drying	Combined pre-drying conditions enhance cassava drying efficiency, yielding moisture content of 6.19% and whiteness index of 92.00. The logarithmic model best describes dehydration kinetics, crucial for tuber processing improvements.	[29]
Ultrasonic Field	Distilled water with ultrasound (DWU), osmotic dehydration with ultrasound (ODU) Frequency of 20 kHz at ultrasound power Pretreatment time of 600 W and 10 min	Hot-air oven drying	Ultrasound pretreatments, especially ODU, significantly reduce drying time of yellow cassava, enhancing effective moisture diffusivity and reducing energy costs. Cavitation effects create microscopic pathways, influencing both internal and external resistances. Parabolic model fits best for DWU, while Page model suits ODU and untreated samples. Thus, ultrasound pretreatment proves beneficial for hot air drying of yellow cassava.	[30]

**Table 2:** Comparison of drying and quality characteristics of drying methods for cassava tuber.

Drying Method	Drying Condition	Drying Characteristics and Quality	References
Sun Drying	Temperatures: 18–25 °C; Humidity: 60–75%.	Sun drying significantly influenced the color, proximate, functional, and pasting properties of cassava flour, impacting its quality and industrial applicability. Despite its simplicity and cost-effectiveness, sun drying demonstrates varying effects on different properties compared to other drying methods, emphasizing its importance in cassava processing.	[71]
Solar Drying	Drying time: 40 h; Average drying temperature: 52 °C.	Solar drying of cassava leads to the formation of coarser particles as a consequence of prolonged drying periods and exposure to light, consequently resulting in a reduced β-carotene content. Additionally, this drying method yields fufu with distinctive texture attributes, thereby affecting its stickiness and softness.	[27]
Hot Air Oven Drying	Load: max 120 kg; Drying time: 660 min; Drying temperature: 50, 60, 70 °C; Fan speed: 0.5, 0.9, and 1.3 m/s.	Hot air oven drying effects on fermented-cooked cassava chips revealed that increasing temperature and fan speed reduced drying time. Effective diffusivity rose with temperature and fan speed, affecting proximate compositions and functional characteristics. Gaussian process regression (GPR)-based modeling proved superior for optimization and control monitoring, vital for product standardization.	[72]
Vacuum Drying	Drying time: 10 h; Drying air temperature 50, 60, and 70 °C; Drying pressure: −60 cmHg.	Vacuum drying significantly influences dried cassava quality. Convective multiple flash drying (CMFD), a novel drying method, achieves desired moisture content in 5–6 h, yielding harder chips compared to convective and vacuum methods. Operating conditions affect moisture content.	[73]
Freeze Drying	Vacuum: Hi MBars pressure, wait for the collector at 22 °C; Run collector: −41 °C for 48 cumulative hours.	Freeze drying significantly preserved color and quality of cassava flour compared to other methods. It yields superior moisture, ash, fat, and protein content, ensuring high-quality flour. Functional properties like swelling power and water absorption are favorable. Pasting properties also indicate its suitability for various food products.	[71]
Fluidized Bed Drying	Drying air temperature: 50, 55, 60, 65, and 70 °C; Solid feed flow rate: 10 g/min and 30 g/min; Airflow rate: 0.012 m^3^/min.	In the continuous vibrated fluidized bed drying of cassava starch, air temperature significantly influences drying, while weir height and solid feed rate have minimal impact. The Page model proves most accurate in describing the drying kinetics.	[74]
Sun Drying and Oven Drying	Sun drying for 4–6 h, followed by oven drying at 55 °C for 24 h.	Drying cassava chips followed by milling into flour reduces hydrogen cyanide (HCN) content by up to 81%, enhancing safety. However, slow drying during rainy seasons can lead to higher acidity, compromising taste and favoring spoilage. Whiteness may be affected by ash content and water quality, influencing product quality.	[75]
Solar-Assisted Heat Pump Drying	Load: 30.8 kg; Drying time: 9 h; Drying temperature: 45 °C; Specific moisture extraction ratio (SMER): 0.38 kg/kWh; Coefficient of performance (COP): 3.38; Thermal efficiency (η): 30.9.	Both solar drying (SD) and solar-assisted heat pump drying (SAHPD) significantly reduced cassava mass and moisture content. SAHPD demonstrated higher drying rate, specific moisture extraction rate, thermal efficiency, and pick-up efficiency compared to SD, indicating its superior performance in drying cassava.	[76]
Hybrid Solar Drying	Load: 300 g Drying time: 3 h Drying temperature: 40, 50, 60 °C	Higher drying temperatures lead to faster and more effective drying of cassava starch. The fastest moisture reduction occurs initially, with the drying process mainly happening during the falling rate period. Hybrid solar dryers, especially when combined with liquefied petroleum gas (LPG), are more effective compared to open sun drying, with an effectivity factor reaching up to 6.4.	[77]
Microwave–Hot Air Drying (MHAD)	Drying temperature: 70 °C; Airflow rate: 1.9 m^3^ min^−1^; Microwave power: 95 W.	MHAD affected the physicochemical properties, drying kinetics, and sensory acceptance of dried cassava. MHAD resulted in similar drying kinetics across cultivars, with no significant differences in moisture removal rates.	[26]

**Table 3:** The utilization of cassava flour in a variety of food products.

Products	Compositions	Key Highlights	References
Biscuits	Cassava flour, full-fat soy-flour, wheat flour, sugar, margarine, egg, baking powder, ginger flour, sugar, margarine and egg	The protein enrichment of cassava flour increased the nutrient content of the biscuits, potentially meeting the nutrient requirements of school children.	[138]
Gluten-free cup cakes	Cassava flour, shortening, egg, milk powder, baking powder and vanilla	The use of cassava flour, along with pumpkin and potato flours, or their mixture, tailored for celiac patients.	[139]
Cookies	Cassava flour, cowpea flour, salt, sugar, skimmed milk, baking fat, and water	A decrease in protein content occurred with the increase in cassava flour substitution, but the addition of cowpea flour led to an overall elevation in protein content.	[140]
Gluten-free flat bread and biscuits	Flat bread: Cassava flour, rice flour, extruded soy protein, xanthan, salt, butter, and water. Biscuits: Cassava flour, rice flour, extruded soy protein, xanthan, sugar, egg, baking powder and butter.	Incorporating cassava flour and extruded soy protein led to an improvement in the nutritional content of both flat bread and biscuits.	[141]
Bread	Cassava flour, wheat flour, fiber, yeast, water, salt and sucrose.	Cassava flour presents a promising solution for replacing up to 30% of wheat flour without significant differences in the final product.	[142]
Bread	Cassava flour, wheat flour, honey, baking fat, yeast, bread improver, water, and salt.	The substitution of sugar with liquid honey in cassava-wheat composite flour formulations led to notable effects on both the pasting properties of cooked dough and the characteristics of the resulting bread.	[143]
Noodles	Cassava flour, water, and alum.	Cassava flour presents a promising solution for replacing wheat flour in noodle manufacturing.	[144]
Noodles	Cassava flour, wheat flour, salt and water.	Noodles made from a composite flour of cassava and wheat showed promising results in terms of acceptability.	[88]

## Data Availability

No new data were created or analyzed in this study. Data sharing is not applicable to this article.
